# Making the invisible audible: a real-world connected speech study in myasthenia gravis

**DOI:** 10.3389/fneur.2026.1756913

**Published:** 2026-05-19

**Authors:** Aurora Zanghì, Paola Sofia Di Filippo, Claudia Rutigliano, Carlo Avolio, Emanuele D’Amico

**Affiliations:** BRAND Center, Department of Medical and Surgical Sciences, University of Foggia, Foggia, Italy

**Keywords:** MG-ADL, myasthenia gravis, patient reported outcomes, quality of life, voice parameters

## Abstract

**Background:**

Bulbar involvement in myasthenia gravis (MG) can affect voice and speech; however, task-dependent and potentially subclinical alterations are often underrecognized.

**Methods:**

We conducted a cross-sectional study of consecutively enrolled adults with confirmed MG at a single center. Participants completed standardized voice recordings and contemporaneous clinical assessments [Myasthenia Gravis–Activities of Daily Living (MG-ADL) and revised Myasthenia Gravis Quality of Life (rMG-QOL15)]. Tasks included sustained vowels, standardized reading, and spontaneous speech. Acoustic metrics comprised root-mean-square intensity (RMS, dB) and fundamental frequency (F0, Hz).

**Results:**

Seventeen patients were enrolled (median age 58 years; 52.9% female). Median MG-ADL was 6.0 and mean rMG-QOL15 was 16.6, with no significant correlation (*ρ* = 0.09, *p* = 0.74). Male participants exhibited significant intensity reductions in connected speech: reading (−3.22 dB, IQR − 5.98 to −2.09; pBH = 0.031; *r* = 0.84) and spontaneous speech (−5.46 dB, IQR − 6.11 to −4.93; pBH = 0.031; *r* = 0.84). Female participants showed smaller, non-significant changes (≤ − 2.2 dB; all pBH > 0.7). F0 shifts were modest and non-significant after correction. Across the cohort, MG-ADL correlated with both RMS and F0 drops in unadjusted models but not after adjusting for sex. rMG-QOL15 showed no overall association, although females demonstrated a correlation between F0 drop and QOL burden (*ρ* = 0.77, *p* = 0.014).

**Conclusion:**

Connected-speech intensity decrements may represent a sensitive acoustic marker of load-dependent and potentially subclinical bulbar dysfunction in MG, not fully captured by patient-reported outcomes, with potential sex-related differences requiring further validation in larger cohorts.

## Introduction

Myasthenia gravis (MG) is an autoimmune disorder characterized by fluctuating skeletal muscle weakness due to impaired neuromuscular transmission (1). Bulbar involvement is common; however, speech and voice alterations may be subtle and underrecognized, including dysphonia, vocal fatigue, reduced loudness, pitch instability, and articulatory imprecision— and may occur even when patients do not report them (1–4). Such alterations may be detectable on clinical examination and can be quantified acoustically, indicating that voice may capture clinically relevant impairment that is underrecognized in routine history-taking.

Voice production integrates respiratory drive, laryngeal valving, and supraglottic articulation (5). Disruption of these systems in neuromuscular disease yields physiologically interpretable acoustic changes, including instability of fundamental frequency (F0) and reduced intensity (6). In MG, prior studies—though small and methodologically heterogeneous—have noted reduced sustained phonation time, declining intensity with repetition, and timing or spectral alterations consistent with fatigability (7–9). However, the use of voice as a practical, quantitative complement to clinical assessment and patient-reported outcomes remains limited. Objective acoustic measures can make otherwise invisible impairments audible, supplementing bedside examination and adding granularity to patient-reported burden captured by instruments such as Myasthenia Gravis Activities of Daily Living scale (MG-ADL) and quality-of-life scales, as QoL15 for MG. This complementary perspective may improve detection and monitoring of bulbar involvement, including load-dependent and potentially subclinical alterations not recognized by patients. In this study we aimed to characterize voice in a cohort of persons with MG (pwMG) using simple, physiologically grounded acoustic features—primarily frequency and intensity—and to examine their alignment with MG-ADL and QoL15 MG as complementary indicators of clinical burden in a real-world observational setting, with a focus on task-dependent and potentially subclinical alteration.

## Methods

### Study design and setting

This cross-sectional observational study was conducted at the BRAND (Breakthrough Research in Autoimmune and Neurodegenerative Diseases) center, University of Foggia (Foggia, Italy).

During a one-month window (1–30 June 2025), participants attended a single index visit at which voice recordings were obtained. At the same visit, MG-ADL and QoL15MG measures were collected contemporaneous with the voice recordings.

Additional clinical and demographic information relevant to the index visit (e.g., diagnosis details, treatment status, ancillary clinical measures) was retrieved retrospectively from the medical record.

### Participants

During the study window, we employed consecutive inclusion of all persons with a confirmed diagnosis of MG, age>18 years and who were admitted to the center and underwent both standardized voice recording and clinical assessment at the same visit.

We excluded persons: (1) with myasthenic crisis within 30 days or major changes in immunotherapy within 30 days; (2) uncontrolled reflux or acute upper airway inflammation within 30 days; (3) primary non-MG laryngeal pathology or other neurologic disorders affecting speech; (4) non-adherence to the recording protocol for severe cognitive impairment.

### Voice recording protocol

Voice recordings were obtained under standardized conditions to ensure comparability across participants and tasks. All sessions were conducted in a quiet clinic room at the BRAND Center, using a fixed setup and procedures. All voice recordings were performed during the outpatient session from 11:00 a.m. onward, after the usual morning dose of pyridostigmine (typically administered between 08:00 and 09:00 a.m.), and without additional dosing prior to testing.

#### Coaching, retrials, and artifact management

Brief, standardized coaching before each item. If plosives, off-axis movement, or coughs occurred within the first 5 s, the recording was stopped and repeated. Maximum two retrials per item to minimize data loss while avoiding selection bias.

#### Calibration and acquisition settings

Input gain was calibrated at the start of each session using a standardized reference phrase and held constant for each participant. The microphone was positioned at a fixed mouth-to-microphone distance of 15 cm and oriented 30° off-axis, with placement verified before each item. Audio was captured using a cardioid condenser microphone (AT2020, Audio-Technica, Tokyo, Japan) mounted in a shock mount and spider, connected via an XLR microphone cable (NSAMB3, Bespeco, Ancona, Italy) to an audio interface (Scarlett series, Focusrite, High Wycombe, United Kingdom) on an anti-vibration stand. An acoustic Reflexion Filter and supplementary panels (sE Electronics–style portable filter or equivalent) were used to reduce room reflections. Monitoring was performed with closed-back headphones (Sennheiser, Wedemark, Germany), and nearfield reference listening was available on studio monitors (ADAM Audio, Berlin, Germany). Recordings were made in Audacity (version 3.7.5; 48 kHz, 24-bit) on a workstation, with all settings held constant across participants.

#### Session protocol

Standardized instructions before each task. Fixed task order across participants and sessions to limit order effects. Procedural deviations annotated in real time; affected segments handled per artifact rules. Segments with evident artifacts (clipping/saturation, impulsive noise, motion) excluded based on *a priori* criteria.

Task sequence: All participants completed, in order, sustained vowel phonation, scripted reading, and brief spontaneous speech.

Sustained vowel: Three repetitions of sustained /a/e/o/ at comfortable pitch and loudness, each for as long as possible without strain, with 30–45 s rest between repetitions. Participants begin after a 1 s count-in, avoid crescendo/decrescendo, and maintain steady output.

Scripted reading: One standardized Italian passage printed in 14-point font, read at a comfortable pace and natural prosody. Participants read a story and are instructed to avoid whispering or exaggerated projection and to pause naturally as needed.

Spontaneous speech: A 90 s response to a neutral prompt (describe yesterday’s activities), encouraging continuous speech without background music or sound effects.

#### Session log and traceability

Structured log includes date/time, room ID, device IDs, interface gain settings, mouth–microphone distance, task order, number of retrials, and notes on relevant events (e.g., coughs, interruptions, ambient noise). Hardware/software settings preserved in preconfigured profiles to ensure reproducibility and quality control across sessions.

#### Quality control and exclusion criteria

Online monitoring via level indicators and operator auditory/visual inspection. Offline review with predefined rejection criteria for saturation/clipping, impulsive noise, positioning deviations, and signal instability. Only artifact-free segments, as defined by *a priori* rules, are retained for outcome analysis.

### Clinical measures and quality of life

The MG-ADL is a patient-reported measure of disease impact composed of eight items, each scored on four levels, yielding a total from 0 to 24; higher totals reflect greater disease burden (10). Scores of 0–1 are commonly interpreted as minimal manifestations, and values of 2 or below have been proposed as a threshold consistent with patient-acceptable symptom state (PASS) (11–13). There is no universally accepted cutoff for moderate or severe activity. Nonetheless, several recent pivotal trials have used MG-ADL thresholds to define active disease: for example, RAISE and REGAIN required MG-ADL scores of at least 6, while ADAPT enrolled patients with scores of 5 or more (14–16).

Health-related quality of life was assessed using the revised MG-QoL15, a 15-item, disease-specific questionnaire with three response options per item (0–2) (17). The total score ranges from 0 to 30, with higher values indicating poorer quality of life, encompassing physical, psychological, and social domains (17). Clinical assessment included the MG-ADL and the QOL15-Myasthenia, ensuring alignment between acoustic and clinical measures for each participant.

In our cohort, treatment exposure was categorized as follows: old immunosuppressor (azathioprine) and immunotherapies (rituximab, eculizumab, ravulizumab, rozanolixizumab); pyridostigmine and steroid therapy were recorded separately.

### Outcomes and covariate definitions

The primary outcomes was to characterize voice parameters in a cohort of pwMG and to examine their alignment with MG-ADL and rQoL15 MG as complementary indicators of clinical burden.

All task-wise acoustics were interpreted against sex-specific normative bands.

Acoustic predictors were derived at the subject level. For the vowel baseline, we computed within-subject medians of fundamental frequency (F0, Hz) and root-mean-square intensity (RMS, dB) across sustained /a/, /e/, and /o/, thereby reducing trial-level variability.

For connected speech, we computed within-subject medians of F0 and RMS separately for reading and spontaneous speech. To capture fatigability, we derived within-subject deltas expressing performance decrement from vowel to connected speech: intensity shifts were defined as rms_shift_reading = RMS(Reading) − RMS(Vowel) and rms_shift_spont = RMS(Spontaneous) − RMS(Vowel); pitch drops were defined as f0_drop_reading = F0(Reading) − F0(Vowel) and f0_drop_spont = F0(Spontaneous) − F0(Vowel).

By construction, negative values indicate that connected speech is softer and/or lower in pitch than sustained vowels for the same individual, consistent with greater task demand and neuromuscular fatigability. This approach captures functional changes that emerge under realistic speaking load and are not always visible in sustained phonation. Age and sex were treated as covariates for adjusted analyses.

We referenced each participant’s F0 to sex-specific normative ranges reported elsewhere (18, 19).

For adult males, typical F0 ranges approximately 85–180 Hz; for adult females, approximately 165–255 Hz. We plotted observed F0 values against these normative bands to visualize deviations by sex. Because absolute sound pressure level depends on the recording chain and calibration, we treated RMS as a relative acoustic level and compared participants’ RMS against reference ranges derived from standardized conversational speech levels reported in the literature (approximately mid-60s to low-70s dB SPL at 1 m in quiet for adult speakers).

### Statistical analysis

Descriptive statistics are presented first to characterize the cohort and task-level acoustics. Continuous variables are summarized as median (interquartile range) alongside mean ± standard deviation, and categorical variables as counts and percentages.

To visualize task- and sex-specific patterns while preserving individual-level information, we employed half-violin plots with SINA overlays and within-subject “spaghetti” trajectories, complemented by sex-specific normative bands, to depict the distribution, central tendency, and load-sensitive variability of F0 and RMS across sustained vowels, reading, and spontaneous speech in pwMG.

We quantified task-related performance changes using a “drop” analysis, defined as the within-subject difference between connected speech and sustained vowels. Specifically, for each participant and metric, we computed Drop = Connected Speech (Reading or Spontaneous Speech) − Vowel. For RMS, negative drops indicate reduced intensity in connected speech relative to vowels; for F0, positive drops indicate higher pitch in connected speech. We visualized the distributions of drops with kernel density estimates by sex and task, and formally tested whether median drops differed from zero using Wilcoxon signed-rank tests with Benjamini–Hochberg correction.

Associations between acoustic predictors (task medians and derived contrasts) and clinical outcomes (MG-ADL and MG-QoL15 totals) were estimated using Spearman’s rank correlation. Because age and sex were available for all participants and represent plausible confounders, adjusted analyses employed partial Spearman correlations controlling for both variables. Two-sided *p*-values are reported without multiplicity correction, with interpretation emphasizing effect sizes, directionality, and consistency across related outcomes. The dataset was complete (no missing values).

Analyses were performed in Python 3.11 using numpy 1.26 (numerics), pandas 2.2 (data management), scipy 1.11 (nonparametric statistics, including Spearman and partial Spearman), statsmodels 0.14 (ancillary inference), and matplotlib 3.8/seaborn 0.13 (visualization). Audio preprocessing and feature aggregation were implemented with reproducible scripts pinned to these versions to ensure deterministic behavior.

## Results

### Descriptive statistics

We enrolled a total cohort of 17 patients, median age 58 years (44.0–72.0), 9 (52.9%) female. Current or former smokers were 5 of 17 (29.4%). Median BMI was 25.2 kg/m^2 (24.7–28.8). Disease duration (DD) was 33.0 months (22.0–153.0) (Table 1).

**Table 1:** Clinical characteristics of the patient cohort.

Variable* *n* = 17	Value
Sex (female)	9 (52.9%)
Age (years)	58.0 [44.0–72.0]
Smokers	5 (29.4%)
Years of instruction	13.0 [8.0–13.0]
BMI (kg/m^2^)	25.18 [24.67–28.8]
Disease duration (months)	33.0 [22–153]
QMG score	5.0 [3.0–12.0]
MG-ADL total	6.0 [5.0–7.0]
AChR antibody status	
*Positive*	11 (64.7%)
*Negative*	6 (35.3%)
MGFA classification	
	IA 3 (17.6%) IIA 4 (23.5%) IIB 9 (52.9%) IIIA 1 (5.9%)
Type of onset	
*Ocular*	3 (17.6%)
*Bulbar*	4 (23.5%)
*Generalized*	9 (52.9%)
N. of pwMG with comorbidities	13 (76.5%)
PwMG on pyridostigmine therapy	11 (64.7%)
PwMG on Steroid therapy	7 (41.2%)
Steroid dose	5.0 [5.0–7.3]
PwMG on Old immunosuppressor*	5 (29.4%)
PwMG on immunotherapies**	5 (29.4%)
N. of pwMG who underwent Thymectomy	6 (35.3%)

Continuous variables are reported as median (Q1–Q3). Binary variables are reported as n (%). N indicates the number of patients. pwMG, persons with Myasthenia Gravis; BMI, Body Mass Index; QMG, Quantitative Myasthenia Gravis score. MG-ADL, Myasthenia Gravis – Activities of Daily Living; AChR, Acetylcholine Receptor; MGFA, Myasthenia Gravis Foundation of America. *Azathioprine; **rituximab, eculizumab, ravulizumab, rozanolixizumab.

Regarding AChR antibody status, 11 patients were positive (64.7%), whilst 6 were negative (35.3%); no patients were MuSK-positive.

MGFA distribution was as follow: IA 3 (17.6%); IIA 4 (23.5%); IIB 9 (52.9%); IIIA 1 (5.9%). Chronic steroid therapy was used in 7(41.2%) pwMG, pwMG on steroid therapy were 7 (41.2%), while pwMG on pyridostigmine therapy were 11 (64.7%). PwMG on old immunosuppressor were 5 (29.4%), and pwMG on immunotherapies were 5 (29.4%) (Table 1).

### MG-ADL and quality of life measured by rMGQoL

**Figure 1:**
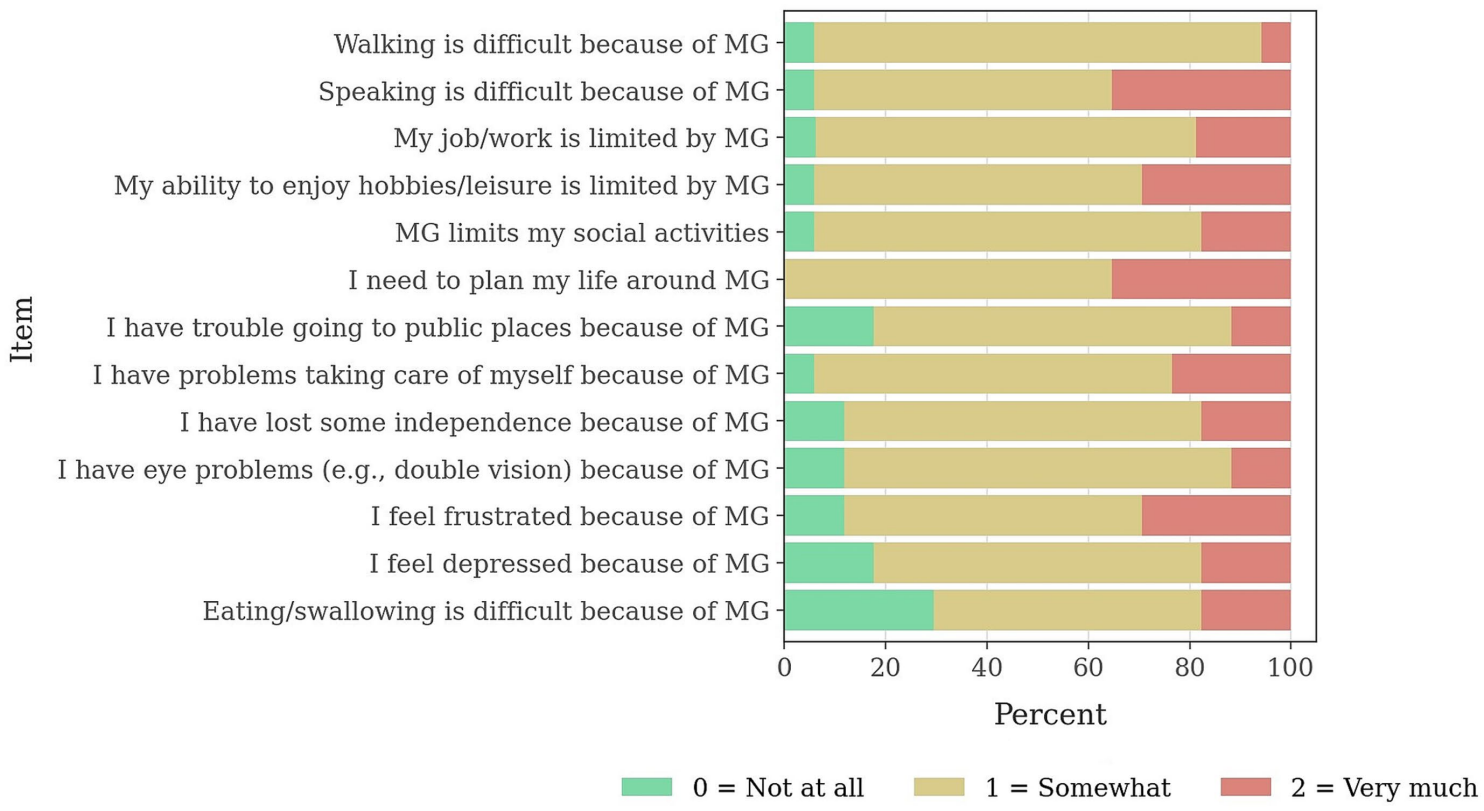
Item-level distributions QoL15 MG. 0 = not all; 1 = somewhat; 2 = so much.

In our cohort, median MG-ADL was 6.0 (5.0–7.0). rMG-QOL15 total scores showed a median of 16.6 (IQR 15.0–18.0), consistent with a moderate burden. Item-level distributions (Figure 1) indicated the highest severe endorsements for planning life around MG (35.3%), eating/swallowing difficulties (35.3%), speaking difficulties (29.4%), low energy/fatigue (29.4%), and difficulty accessing public places (23.5%). The lowest severe endorsements were observed for eye problems such as double vision (11.8%), depressive feelings (11.8%), and difficulties with personal grooming/self-care (5.9%).

**Figure 2:**
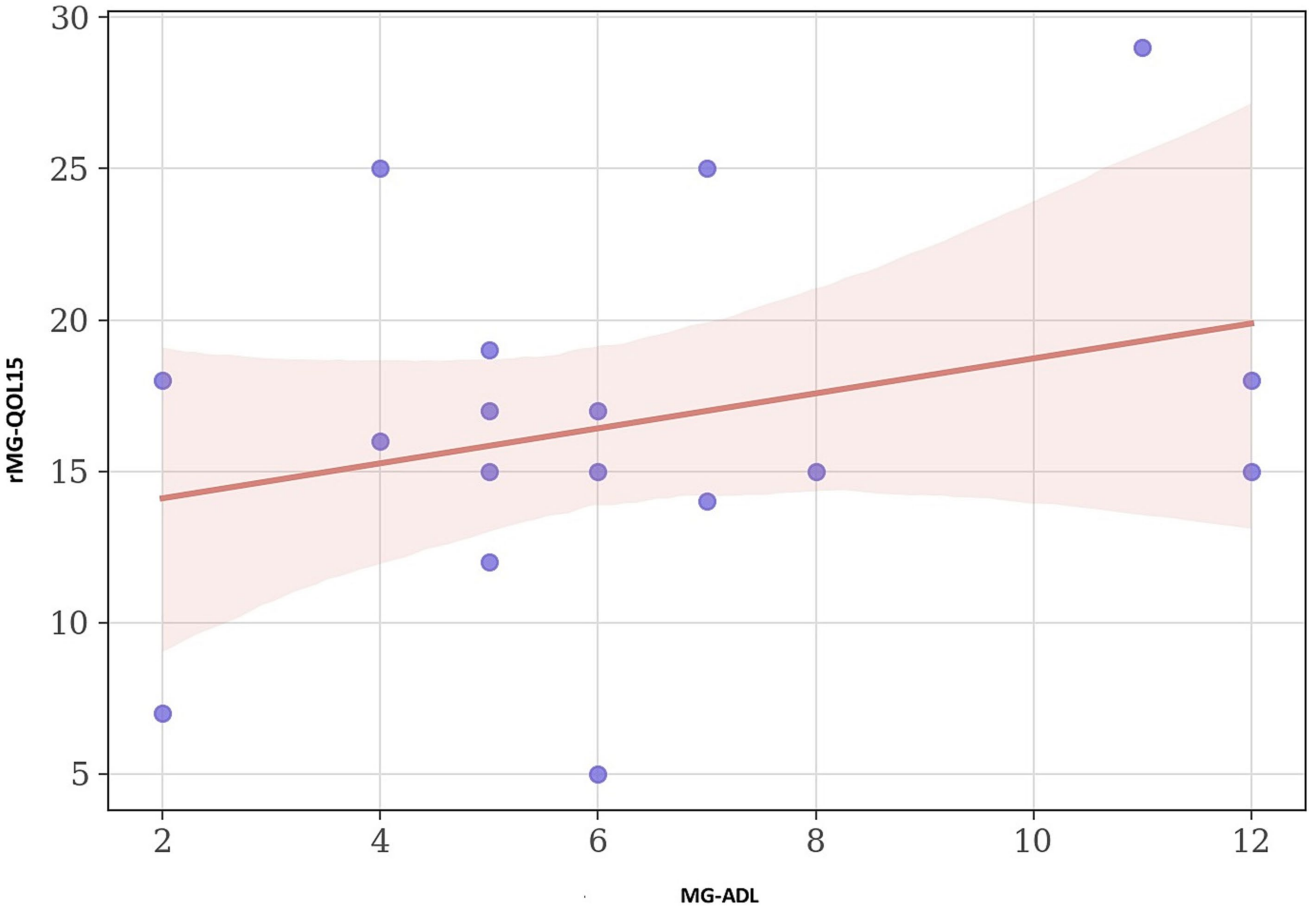
Association Between MG-ADL and MG-QOL15 Scores in Myasthenia Gravis. Scatter plot of MG-ADL versus MG-QOL15 total scores. Points represent individuals; red line is the linear regression with 95% CI.

rMG-QOL15 total was not associated with MG-ADL (Spearman *r* = 0.09, *p* = 0.74) (Figure 2).

### Voice analysis

The voice analysis for single task showed that pwMG showed preserved sex-related pitch separation with a clear intensity deficit that was larger in females and most pronounced in connected speech (Supplementary Table 1).

**Figure 3:**
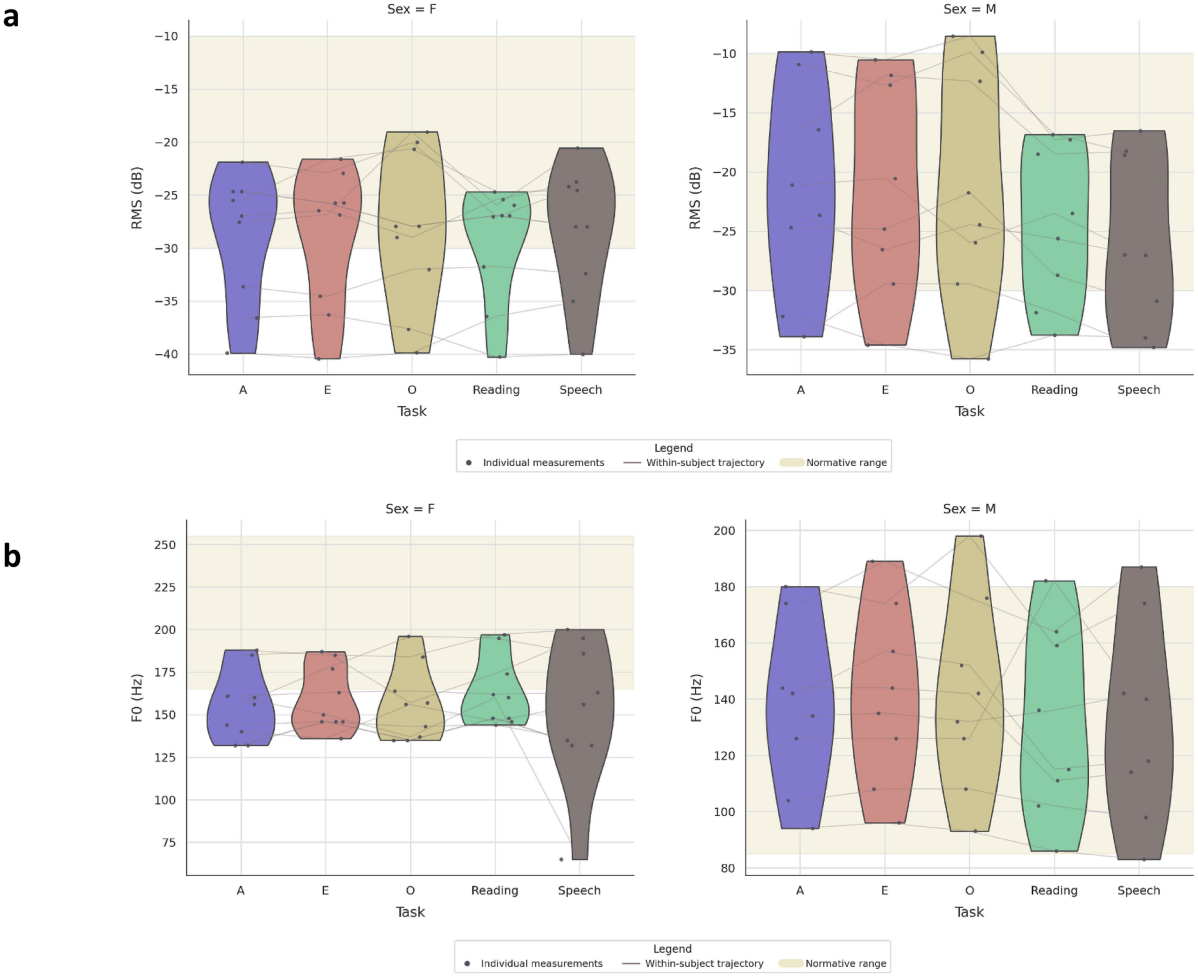
Task- and sex-specific voice intensity (RMS) distributions with individual observations and within-subject trajectories. Half-violin plots show the distribution of **(a)** RMS (dB) across tasks (A, E, O, Reading, Speech) stratified by sex; SINA-style points display all individual measurements, and thin gray lines connect repeated measures within the same subject across tasks. A pale band denotes the illustrative normative RMS range for this recording setup. **(b)** F0 (Hz) across tasks, faceted by sex; SINA-style points represent all observations, and thin gray lines indicate within-subject changes across tasks. The shaded band marks sex-specific normative ranges (Male 85–180 Hz; Female 165–255 Hz).

Figure 3 shows task- and sex-stratified distributions with all individual observations and within-subject trajectories, demonstrating that voice intensity (RMS) remains centered within the illustrative −30 to −10 dB band but exhibits task-dependent dispersion that enlarges and develops lower tails in connected speech (Reading/Speech) compared with sustained vowels (A/E/O), and fundamental frequency (F0) remains broadly within sex-specific normative ranges (Male 85–180 Hz; Female 165–255 Hz), with tighter dispersion for vowels and expanded variance under connected-speech demands; within-subject overlays highlight modest RMS decrements and increased instability from vowels to connected speech and relatively stable F0 across vowels with larger idiosyncratic shifts during connected speech, indicating load-sensitive variability that widens dispersion more than shifting central tendency.

**Figure 4:**
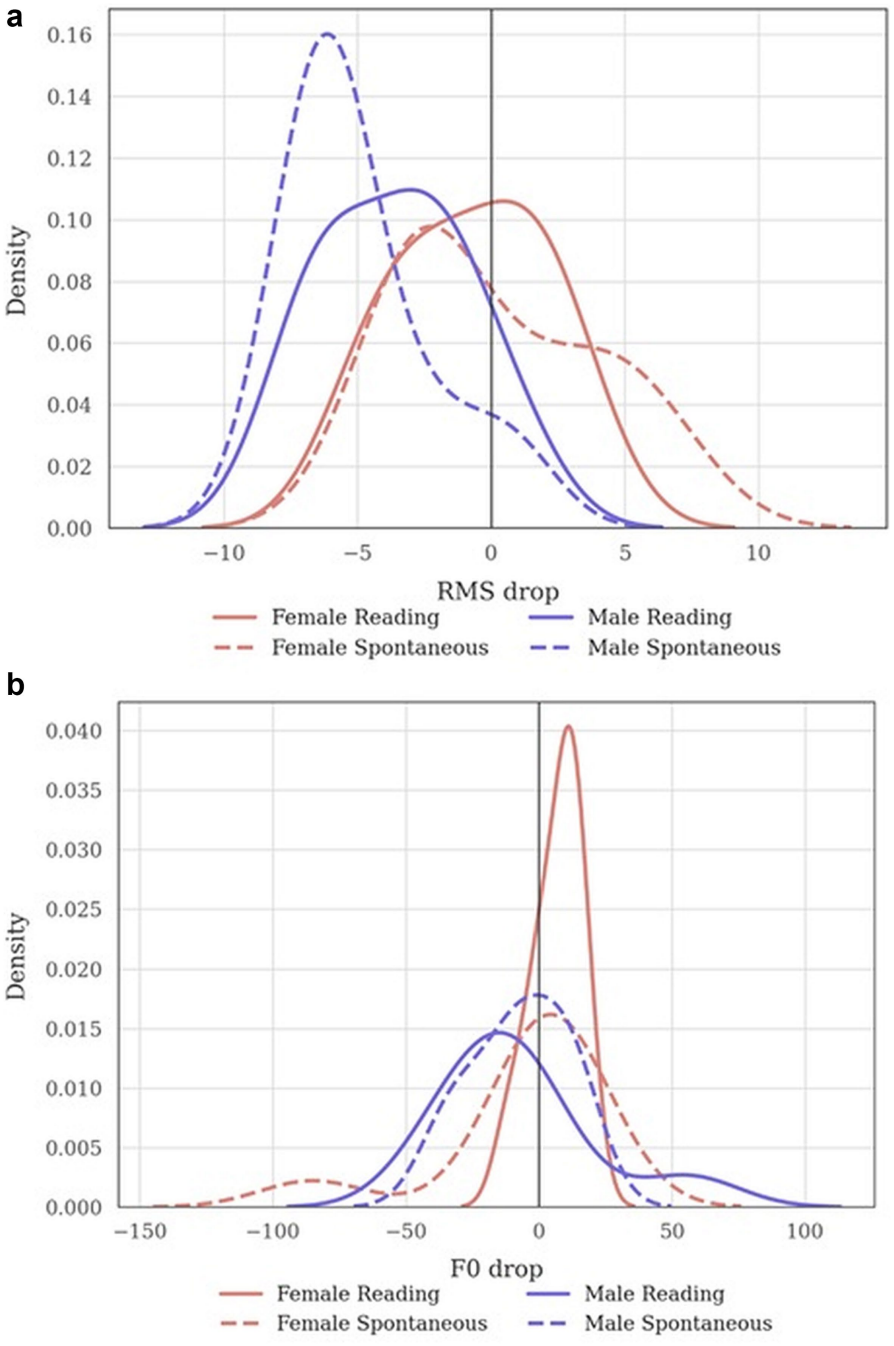
Connected Speech minus Vowel (Density). **(a)** RMS change: Kernel density of RMS change computed as Connected Speech minus Vowel; negative values indicate that connected speech is softer than sustained vowels. Reading is shown with a solid line, Spontaneous Speech with a dashed line. Female participants are shown in coral, Male participants in slate blue. A vertical reference line marks zero change. **(b)** F0 change: Kernel density of F0 change computed as Connected Speech minus Vowel; negative values indicate lower pitch in connected speech; positive values indicate higher pitch. Reading is shown with a solid line, Spontaneous Speech with a dashed line. Female participants are shown in coral, Male participants in slate blue. A vertical reference line marks zero change.

Task related performance with drop analysis revealed that connected speech was softer than sustained vowels in males, with consistent and clinically meaningful reductions in RMS (Table 2 and Figure 4). In reading, the median decrease was −3.22 dB (IQR − 5.98 to −2.09; Wilcoxon *p* = 0.0156; BH *p* = 0.0312; large effect size *r* ≈ 0.84; *n* = 8). Spontaneous Speech showed an even larger decline (approximately −5 to −6 dB), also significant with a large effect; see Table 2 for exact coefficients and confidence summaries. In contrast, females exhibited smaller and non-significant intensity changes: the median RMS drop in Reading was −1.17 dB (IQR − 3.09 to 1.52; BH *p* = 0.760; *n* = 9), and in Spontaneous Speech −2.22 dB (IQR − 2.30 to 3.20; BH *p* = 0.910; *n* = 9). These patterns are mirrored by the density plots, which for males show a leftward shift below zero for both connected-speech tasks, whereas female curves cluster near zero.

**Table 2:** Wilcoxon signed-rank test results for density drop measurements by sex, task, and metric.

Sex	Task	Metric	*W*	*p*-value	z-score	Effect size (r)	Median	Q1	Q3	*p*-value (BH corrected)
Female	Reading	RMS	17.0	0.570	0.652	0.217	−1.167	−3.093	1.522	0.760
Female	Reading	F0	5.0	0.068	1.718	0.573	10.000	0.000	13.000	0.273
Female	Spontaneous speech	RMS	21.0	0.910	0.178	0.059	−2.223	−2.302	3.201	0.910
Female	Spontaneous speech	F0	14.5	0.623	0.355	0.118	0.000	−3.000	10.000	0.623
Male	Reading	RMS	1.0	0.016*	2.380	0.842	−3.219	−5.975	−2.091	0.031*
Male	Reading	F0	9.0	0.250	1.260	0.446	−10.000	−24.000	−4.000	0.500
Male	Spontaneous speech	RMS	1.0	0.016*	2.380	0.842	−5.901	−6.325	−4.657	0.031*
Male	Spontaneous speech	F0	12.5	0.461	0.770	0.272	−8.000	−15.750	8.750	0.615

W, Wilcoxon test statistic; Q1, first quartile; Q3, third quartile; BH, Benjamini-Hochberg correction for multiple comparisons; RMS, root mean square; F0, fundamental frequency. *p < 0.05 after correction for multiple comparisons.

Pitch (F0) differences from vowels to connected speech were modest and not robust after multiple-comparison correction in either sex (Table 2). Females showed a tendency toward slightly higher pitch in Reading (median +10 Hz; IQR 0–13; Wilcoxon *p* = 0.068; BH *p* = 0.273; *r* ≈ 0.57; *n* = 9) and negligible change in Spontaneous Speech (median 0 Hz; IQR − 3 to 10; BH *p* = 0.623; *n* = 9). In males, Reading showed a small downward shift (median −10 Hz; IQR − 24 to −4; BH *p* = 0.50; *n* = 8), with Spontaneous Speech centered around zero and non-significant. Taken together, the data indicate that functional degradation from vowels to connected speech is primarily expressed as reduced intensity (RMS) in males, with minimal and non-robust pitch adjustments across groups.

### Association between voice parameters and MG-ADL

In the primary analysis across all subjects, higher MG-ADL scores were associated with more positive acoustic drops (Reading − Vowel) in both intensity and pitch. RMS_drop: *β* = 0.54, *p* = 0.011; F0_drop: *β* = 2.56 Hz, *p* = 0.030 (Supplementary Figures 1, 2).

Results remained directionally consistent after adjusting for sex (RMS *β* = 0.40, *p* = 0.163; F0 *β* = 1.82 Hz, *p* = 0.225) and within sex-stratified analyses. A formal MG-ADL × sex interaction test was not significant, supporting a single overall effect. Sex-stratified estimates are reported in Supplementary Figures 1, 2.

### Association between voice parameters and quality of life measured by 15 MgQoL

In the combined sample, QOL-15 total score showed no significant association with RMS drop (reading − vowel) or F0 drop (Supplementary Table 2 and Supplementary Figure 3). When stratified by sex, correlations remained non-significant among males. Among females, F0 drop demonstrated a moderate positive association with QOL-15 total (Pearson *r* ≈ 0.70, *p* ≈ 0.036; Spearman *ρ* ≈ 0.77, *p* ≈ 0.014), whereas RMS drop was not associated. These findings suggest that greater perceived disease impact (higher QOL-15 total) is specifically related to larger reading–vowel F0 differences in females, while RMS intensity changes do not track QOL-15 across strata.

## Discussion

This study shows that brief, standardized voice recordings can capture clinically meaningful, task-dependent and potentially subclinical aspects of bulbar dysfunction in MG using a simple contrast between connected speech and sustained vowels. Acoustic indices derived from this contrast relate to function as quantified by MG-ADL in the overall cohort, supporting voice as a pragmatic, objective marker of everyday impairment rather than a proxy for general wellbeing. The observation that MG-ADL associations attenuate in sex-stratified analyses likely reflects reduced power rather than a lack of effect, given the directionally consistent patterns and the stable findings in the combined sample. In parallel, we observed distinct sex-related profiles that inform interpretation and clinical deployment. Women tended to remain close to the lower limit of the normative band for speech measures even when clinically compensated, whereas men exhibited relatively larger performance losses under connected-speech demands. These complementary patterns suggest that sex-aware interpretation may be warranted: proximity to the lower-normal boundary in women should not be discounted, and relatively greater connected-speech decrement in men may unmask deficits that are less apparent during sustained phonation. However, these differences should not be interpreted as reflecting differences in disease severity, but rather as potentially related to physiological and acoustic differences in voice production. The greater robustness of RMS compared to F0 likely reflects their distinct physiological determinants, with vocal intensity being more directly dependent on respiratory support and phonatory effort, whereas F0 is influenced by more complex and variable laryngeal control mechanisms, making it less consistent across tasks.

Taken together, these findings may have relevant implications for clinical interpretation. Because connected-speech metrics relate to MG-ADL, serial voice recordings offer an avenue to objectify symptoms that are often “invisible” in routine history-taking and difficult to quantify at the bedside (20, 21). Repeated measurement can establish a personalized baseline and detect deviations that may prompt therapeutic reassessment, rehabilitation targeting, or closer follow-up. In practice, pairing a brief reading passage with a sustained vowel imposes minimal burden, is feasible for in-clinic and at-home monitoring, and can be standardized sufficiently to support longitudinal decision-making. Importantly, sex-specific reference ranges and alert thresholds should be incorporated into any clinical workflow to mitigate misclassification at the margins.

These findings align with previous observations of dysphonia, vocal fatigability, pitch instability, reduced loudness, and articulatory imprecision in MG, although such features have often been documented only anecdotally rather than systematically (2–4, 7, 9, 13, 22). More generally, acoustic perturbation and intensity-related metrics can index neuromuscular inefficiency, and that task demands are known to modulate dysphonia in related bulbar disorders (22). However, systematic use of a connected-speech versus sustained-vowel contrast as a compact, task-dependent biomarker, evaluated across sessions and explicitly linked to MG-ADL, has been limited. Prior studies have emphasized either sustained vowels or broader speech tasks without explicitly operationalizing their difference as a biomarker and without integrating sex-specific interpretation. In this context, the present work adds three elements: a direct, standardized contrast between connected speech and sustained phonation; evidence that this contrast relates to MG-ADL in the overall sample; and a clear, clinically interpretable sex pattern that supports sex-aware thresholds. To our knowledge, this combination of design, analysis, and clinical linkage has not been reported as a unified framework, positioning the study as an advance over existing literature rather than a replication of prior approaches. These findings should be interpreted in light of the study design, which is not intended to detect overt bulbar dysfunction per se, but rather to capture load-dependent and potentially subclinical alterations in speech performance through a within-subject, task-based paradigm. Although the QOL-15 results were not significant in the combined sample and showed an association with F0 only among females, this divergence from MG-ADL is not unexpected. Quality-of-life instruments capture a broad construct that includes psychosocial and systemic factors beyond bulbar function, and they need not track tightly with voice-specific impairments (23).

Importantly, treatment exposure in this cohort reflects real-world management strategies, including treatment optimization and steroid-sparing approaches, and should not be interpreted as a direct surrogate of disease severity.

The present data therefore emphasize the value of pairing objective acoustics with function-facing scales such as MG-ADL, while encouraging incorporation of voice-focused instruments and clinician-rated bulbar severity in future work to refine construct validity (24).

Several limitations should be considered when interpreting these findings. Sex-stratified analyses were underpowered for some effects; therefore, observed differences should be interpreted cautiously and not as evidence of differential disease severity. Future studies with larger, balanced cohorts will be essential to consolidate effect sizes and to derive stable sex-specific thresholds. The absence of a healthy control group represents a limitation of the present study, and future investigations including control populations will be necessary to further establish the specificity of this paradigm.

Despite standardized protocols, residual confounding by medication timing, circadian fluctuation, and recording environment cannot be fully excluded; future designs should incorporate protocolized capture relative to dosing and day time, along with device calibration. The current analyses establish cross-sectional validity and session-level stability, but prospective evaluation of responsiveness to treatment changes, exacerbations, and recovery remains a priority. Generalizability to more severe bulbar phenotypes, older populations, and multilingual speakers also warrants confirmation.

In summary, a brief, paired voice protocol yields task-dependent acoustic measures that align with functional impairment in MG and highlight potentially relevant sex-related patterns. The approach may complement routine assessment by objectifying symptoms that are often underreported, enabling longitudinal monitoring with minimal burden, and supporting sex-aware interpretation. In the context of prior literature, which has predominantly emphasized sustained vowels or unstructured speech measures without a standardized contrast or a direct link to MG-ADL, this work provides a pragmatic framework for voice-based monitoring in MG and supports further validation in larger, longitudinal cohorts.

## Data Availability

The raw data supporting the conclusions of this article will be made available by the authors, without undue reservation.

## Supplementary material

Supplementary Figure 1 Kernel density of RMS in the whole cohort and by sex (density of raw values). Kernel density of raw RMS values by task and sex. Vowel is dotted, Reading solid, Spontaneous Speech dashed. Female participants are shown in coral, Male participants in slate blue. Line styles distinguish tasks to allow immediate comparison of task-dependent loudness profiles. **(a)** RMS, Whole cohort; **(b)** RMS, female; **(c)** RMS, male.

Supplementary Figure 2 Kernel density of F0 in the whole cohort and by sex (density of raw values). Kernel density of raw F0 values by task and sex. Vowel is dotted, Reading solid, Spontaneous Speech dashed. Female participants are shown in coral, Male participants in slate blue. Line styles distinguish tasks to visualize task-dependent pitch profiles. **(a)** F0, Whole cohort; **(b)** F0, female; **(c)** F0, male.

Supplementary Figure 3 rQOL-MGADL-15 versus RMS drop and F0 drop (reading—vowel) stratified by sex. Upper panel: rQOL-MGADL 15 versus RMS drop Scatter points represent individual participants; lines depict sex-specific least-squares regression with 95% confidence band. Lower panel: rQOL-MGADL vs F0 drop Scatter points represent individual participants; lines depict sex-specific least-squares regression with 95% confidence band.
