# Supplementary figures and images for: Making the invisible audible: a real-world connected speech study in myasthenia gravis
Source: Front Neurol. 2026 May 19;17:1756913. doi: 10.3389/fneur.2026.1756913 (PMC13226019; doi:10.3389/fneur.2026.1756913)

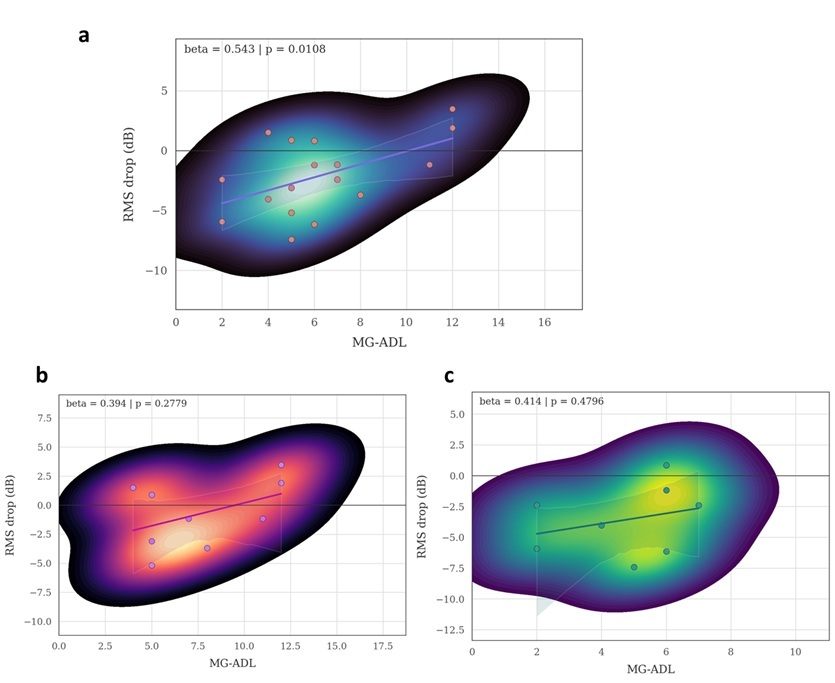

Supplement: Supplementary Figure 1 — Kernel density of RMS in the whole cohort and by sex (density of raw values). Kernel density of raw RMS values by task and sex. Vowel is dotted, Reading solid, Spontaneous Speech dashed. Female participants are shown in coral, Male participants in slate blue. Line styles distinguish tasks to allow immediate comparison of task-dependent loudness profiles. (a) RMS, Whole cohort; (b) RMS, female; (c) RMS, male. [file Image_1.tiff]

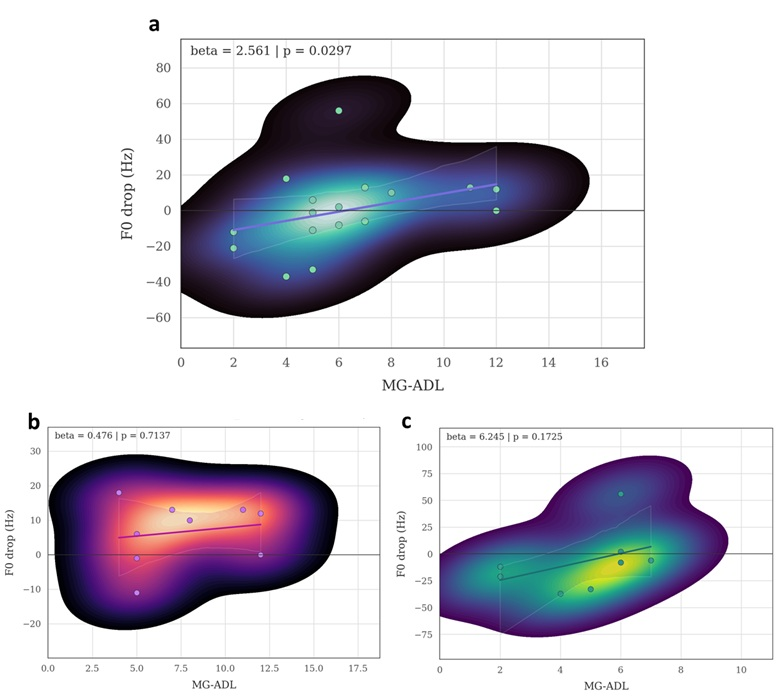

Supplement: Supplementary Figure 2 — Kernel density of F0 in the whole cohort and by sex (density of raw values). Kernel density of raw F0 values by task and sex. Vowel is dotted, Reading solid, Spontaneous Speech dashed. Female participants are shown in coral, Male participants in slate blue. Line styles distinguish tasks to visualize task-dependent pitch profiles. (a) F0, Whole cohort; (b) F0, female; (c) F0, male. [file Image_2.tiff]

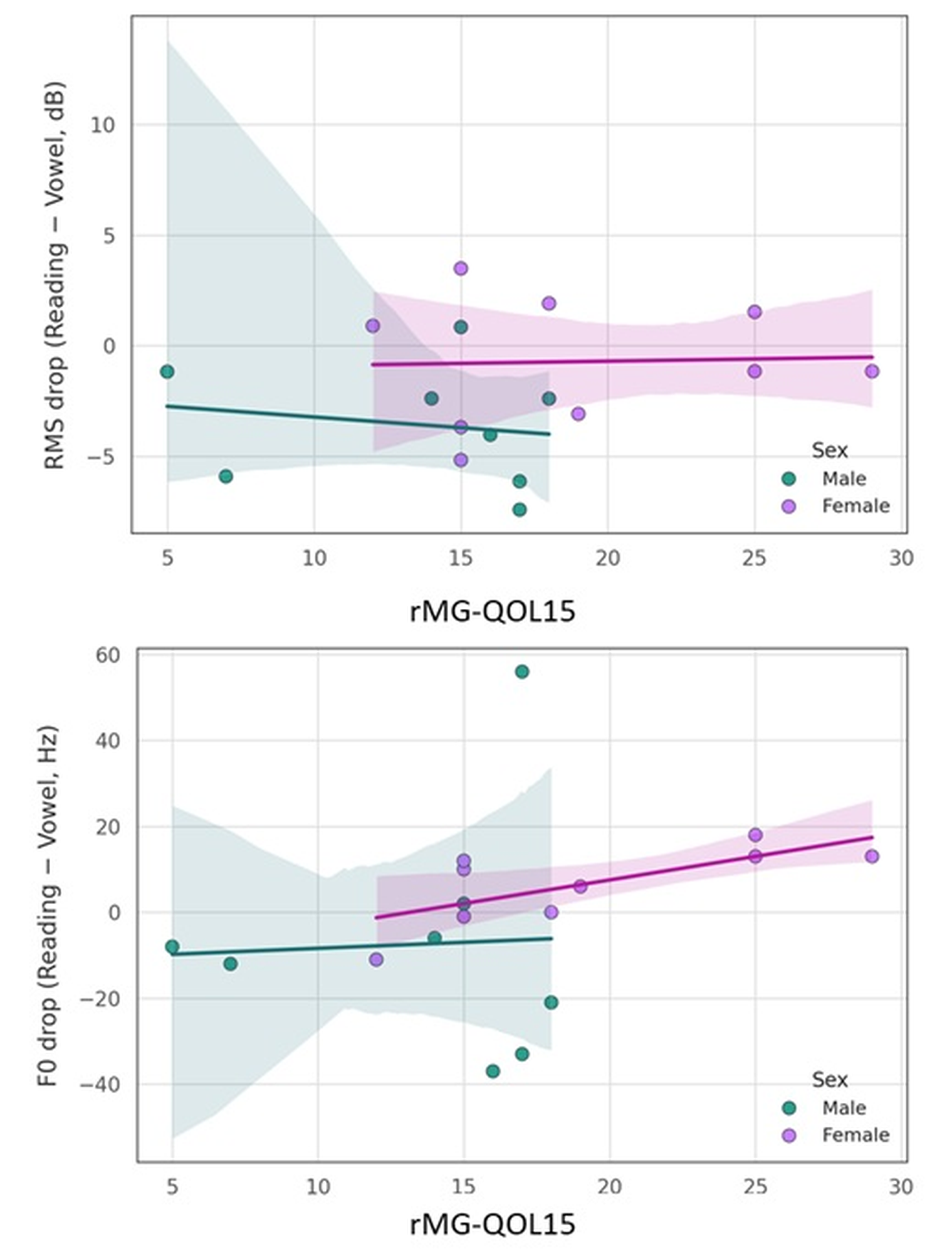

Supplement: Supplementary Figure 3 — rQOL-MGADL-15 versus RMS drop and F0 drop (reading—vowel) stratified by sex. Upper panel: rQOL-MGADL 15 versus RMS drop Scatter points represent individual participants; lines depict sex-specific least-squares regression with 95% confidence band. Lower panel: rQOL-MGADL vs F0 drop Scatter points represent individual participants; lines depict sex-specific least-squares regression with 95% confidence band. [file Image_3.tiff]
