# Supplementary material for: Making the invisible audible: a real-world connected speech study in myasthenia gravis
Source: Front Neurol. 2026 May 19;17:1756913. doi: 10.3389/fneur.2026.1756913 (PMC13226019; doi:10.3389/fneur.2026.1756913)
Supplement: Supplementary file 4 [file Table_1.pdf]

Supplementary Table 1.

Voice analysis for single task

**Variable**	**Total**<br>**n=17**	**Female**<br>**n=9**	**Male**<br>**n=8**
**Sound A**			
Fundamental Frequency f0 [Hz]	144 [132 – 161]	138[120.5, 151.5]	156.0 [140.0, 161.0]
Peak f0 Achieved	0.007 [0.006 – 0.007]	0.007 [0.007, 0.009]	0.006 [0.006, 0.006]
RMS [dB]	-24.7 [-32.2 – -21.9]	-22.4 [-26.6, -15.0]	-27.0 [-33.7, -24.7]
**Sound E**			
Fundamental Frequency f0 [Hz]	149 [137 – 170]	139.5 [121.5, 161.2]	150.0 [146.0, 177.0]
Peak f0 Achieved	0.007 [0.006 – 0.008]	0.007 [0.006, 0.008]	0.005 [0.005, 0.006]
RMS [dB]	-23.90 [-30.85 – -21.50]	-22.7 [-27.3, -12.5]	-26.5 [-34.5, -25.8]
**Sound O**			
Fundamental Freuquency f0 [Hz]	140.5 [129 – 160]	137 [121.5, 158]	156[137.0, 164.0]

Peak f0 Achieved	0.007 [0.006 – 0.008]	0.007 [0.007, 0.008]	0.007 [0.005, 0.009]
RMS [dB]	-24.10 [-31.20 – -21.70]	-23.1 [-26.8, -11.7]	-27.9 [-32.0, -20.7]
**Reading**			
Fundamental Frequency f0 [Hz]	148 [136 – 164]	125.5 [108.8, 160.2]	160.0 [148, 174]
Peak f0 Achieved	0.007 [0.005 – 0.009]	0.009 [0.007, 0.010]	0.005 [0.005, 0.006]
Duration [s]	27.80 [21.40 – 33.60]	30.0 [21.6, 55.8]	40.0 [37.0, 46.0]
RMS [dB]	-20.30 [-24.80 – -18.10]	-27.0 [-31.7, -18.5]	-28.0 [-32.4, -24.2]
Number of Pauses	12 [8 – 17]	8 [5, 9.5]	6 [3, 7]
**Spontaneous Speech**			
Fundamental Frequency f0 [Hz]	140 [118 – 174]	129 [110, 150]	156 [132, 186]
Peak f0 Achieved	0.007 [0.006 – 0.009]	0.008 [0.007, 0.009]	0.006 [0.006, 0.008]
RMS [dB]	-21.70 [-26.10 – -19.40]	-24.6 [-29.5, -18.2]	-26.9 [-31.7, -26.0]

Number of Pauses	10 [6 – 14]	14 [11.8, 16.2]	12 [8, 13]
