# Supplementary material for: Making the invisible audible: a real-world connected speech study in myasthenia gravis
Source: Front Neurol. 2026 May 19;17:1756913. doi: 10.3389/fneur.2026.1756913 (PMC13226019; doi:10.3389/fneur.2026.1756913)
Supplement: Supplementary file 5 [file Table_2.docx]

**Sample Characteristics and Quality of Life Analysis (MG-QOL15)**

| **Variable** | **Value** | **p-value** |
| --- | --- | --- |
| **Correlations^***^** |  |  |
| MG-QOL15 vs MG-ADL (Spearman's r) | 0.13 | 0.622 |
| **Comparisons by clinical subtype** |  |  |
| Bulbar vs non-bulbar symptoms* | 15.0 vs 13.0 | 0.818 |
| Ocular vs non-ocular symptoms* | 13.0 vs 15.0 | 0.482 |
| Generalized vs non-generalized form* | 15.0 vs 13.0 | 0.884 |
| **Comparisons by treatment** |  |  |
| Steroid therapy vs control* | 15.0 vs 13.0 | 0.138 |
| New therapy vs control* | 16.0 vs 13.0 | 0.069† |

*** sex and age adjusted

*Values expressed as medians; comparisons performed using Mann-Whitney test

†Trend toward statistical significance (p < 0.10)

rMG-QOL15: Myasthenia Gravis Quality of Life 15-item scale (range 0-30, higher scores indicate worse quality of life)

MG-ADL: Myasthenia Gravis Activities of Daily Living scale
